# Dataset on improved nutritional quality and safety of grilled marinated and unmarinated ruminant meat using novel unfiltered beer-based marinades

**DOI:** 10.1016/j.dib.2019.104801

**Published:** 2019-11-15

**Authors:** Charles F. Manful, Natalia P. Vidal, Thu H. Pham, Muhammad Nadeem, Evan Wheeler, Melissa C. Hamilton, Karen M. Doody, Raymond H. Thomas

**Affiliations:** School of Science and the Environment/ Boreal Ecosystem Research Initiative, Grenfell Campus, Memorial University of Newfoundland, Corner Brook, A2H 5G4, Canada

**Keywords:** Cervid meat, Fatty acid composition of grilled beef and moose meat, Novel unfiltered beer-based marinades, Lipid oxidation and conjugated fatty acids, Dietary antioxidants and polyphenols, Gas chromatography/mass spectrometry

## Abstract

Objective of this data isn brief article is to present the associated data set regarding the revised article entitled “Unfiltered beer based marinades reduced exposure to carcinogens and suppressed conjugated fatty acid oxidation in grilled meats” recommended for publication in *Food Control* [1]. Grill food safety and quality is a major concern globally. Here in we present data demonstrating the use of novel unfiltered beer based marinades in improving the nutritional quality and safety of grilled ruminant meat. Grilling can lead to the formation of harmful compounds and modify the functional lipids in meats via oxidation, thereby affecting the nutritional quality and safety of the finished product. Lipid oxidation is a deteriorative process involving the degradation of lipid double bonds and the formation of new compounds. Some of these compounds can result in reduced meat quality and off-flavours affecting the sensory, nutritional quality and safety of grilled meat. Unfiltered beers, herbs and spices are known to be excellent sources of antioxidants and polyphenols which can suppress oxidation of functional lipids in grilled meat. Novel unfiltered beer based marinades were developed and used to marinate ruminant meat (beef and moose) prior to grilling. The effect of marination on the fatty acid profile, including saturated, mono- and polyunsaturated fatty acids, of grilled meat was analyzed by gas chromatography/mass spectrometry (GC/MS). In this data in brief article, we include 3 tables containing the fatty acid composition of unmarinated and marinated grilled ruminant meats (beef and moose), a figure showing the percent distribution of grilled meat fatty acid classes, and 2 figures on Pearson's correlation for the associations between phenolic contents, oxidation status and total conjugated linolenic acid (CLA) content. To the best of our knowledge, there is a paucity of information in the literature on the fatty acid composition of wild Cervid meat following preparation by grilling. Grill food safety and nutritional quality is of significant interest to researchers and consumers in the scientific and general food science communities. This article provides data on the fatty composition of grilled moose meat and could be of value to fill the paucity of information currently available in the scientific community on the observed fatty acid composition of grill moose meat. Furthermore, the article presents data on the effects of beer based marinade formulations on the quality of the fatty acid composition of grilled ruminant meats (beef and moose). The growing awareness of the benefits of dietary fatty acids in enhancing personal and population health by reducing the risk factors for cardiovascular diseases and neurodegenerative disorders means that consumers demand meat products with improved fatty acid composition [2,3]. Cervids such as moose (Alces alces) are popular as superior sources of low-fat lean meat with balanced omega 6:3 essential fatty acids compared to traditional farm raised or domesticated meat animals due to the forage they consume as a normal part of their diet [2,4,5]. Furthermore, session ale beers is currently a global phenomenon derived from unique combinations of grains, hops, fruits and herbs to produce low alcohol by volume beers with unique flavors popular among consumers. Two unfiltered session beers were used as base ingredients to produce two novel marinades infused with unique combination of antioxidant rich herbs and spices as a suitable system for the production of grilled foods with enhanced nutritional and sensory characteristics. Whilst there are a limited number of studies in the literature that have used unfiltered beers to evaluate the effects of these beers on suppression of lipid oxidation in grilled meat, none to the best of our knowledge has evaluated the effect of antioxidant rich unfiltered beer based marinades on fatty acid composition of grilled meat systems [6] [7]. As such, this data set presents the concept of using craft beers (specifically session ales) infused with unique combination of herbs and spices to produce unfiltered beer base marinades with enhanced ability to improve grill food sensory attributes and quality, and demonstrates that novel formulations of popular unfiltered India session ale and wheat ale based marinades infused with unique combinations of herbs and spices could be used to marinate beef and moose meats prior to grilling to preserve meat lipids including anticarcinogenic linoleic acid and essential ω3 and ω6 fatty acids.

**Table:** Specifications Table

Subject	Agricultural and Biological Sciences
Specific subject area	Food Science
Type of data	Figures Tables
How data were acquired	Data were acquired by the extraction of meat lipids from marinated and unmarinated grilled moose and beef meat and subsequent analysis as fatty acid methyl esters (FAMEs) by GC/MS. Fatty acids detected in the samples were identified and quantified using the following standards: a 37 Fatty acid standard mix, conjugated linoleic acid (CLA) methyl ester mix and conjugated linolenic acid (CLN) methyl ester mix (Supelco, Bellefonte, PA). Fatty acids concentration is expressed as mg/g meat
Data format	Raw Analyzed
Parameters for data collection	Meat lipids were extracted from marinated and unmarinated grilled moose and beef meat by Bligh and Dyer method [8]. Extracted lipids were methylated under acidic conditions using methanolic HCL at 60 °C for 80 min, and the resulting FAMEs composition was analyzed on a Trace 1300 gas chromatograph coupled to a TSQ 8000 Triple Quadrupole mass spectrometer (Thermo Scientific, Brampton, ON, Canada). Fatty acids detected in the meat samples were quantified using standard curves based on authenticated lipid standards. Individual fatty acid contents were expressed on mg/g meat basis. Pearson's correlation tests between total CLA, total phenolic content, and total oxidation status of the grilled meat samples were performed using XLSTAT Premium version (Addinsoft, New York, USA).
Description of data collection	Three replicates (n = 3) were employed per experimental treatment. One-way analysis of variance (ANOVA) was used to determine if there were significant differences between the fatty acid contents observed in marinated and unmarinated moose and beef samples. Where treatment effects were significant, the means were compared with Fisher's Least Significant Difference (LSD), α = 0.05.
Data source location	Memorial University of Newfoundland, Corner Brook, Newfoundland, Canada
Data accessibility	Raw data are available within this article as supplementary material
Related research article	Charles F. Manful*, Natalia P. Vidal, Thu H. Pham, Muhammad Nadeem, Evan Wheeler, Melissa C. Hamilton, Karen M. Doody, Raymond H. Thomas* Unfiltered beer based marinades reduced exposure to carcinogens and suppressed conjugated fatty acid oxidation in grilled meats. Food Control

**Table:** 

**Value of the Data** • Data set consists of fatty acid composition of grilled marinated and unmarinated beef and moose meat useful to determine the effects of grilling and antioxidant rich unfiltered beer based marinades on the nutritional quality of the fatty acid composition of grilled ruminant meats • The data demonstrates the application of novel unfiltered beer based marinades composed of antioxidant rich unfiltered beers, herbs and spices to supress oxidative degradation of functional lipids including ω3 and ω6 fatty acids in grilled ruminant meats. • The data set and marinade ingredients could be used as a reference to formulate new unfiltered beers infused with different combination of herbs and spices, for development of craft beer based marinades to produce a potentially healthier and safer meat product on the grill. • Significantly, the data demonstrates that unfiltered beer based marination preserved anticarcinogenic conjugated linoleic acids (CLAs) in moose and beef from oxidative degradation during grilling, and could be a useful cooking strategy to preserve CLAs and reduce cancer risks associated with red meat consumption.

## 1 Data

**Fig. 1:**
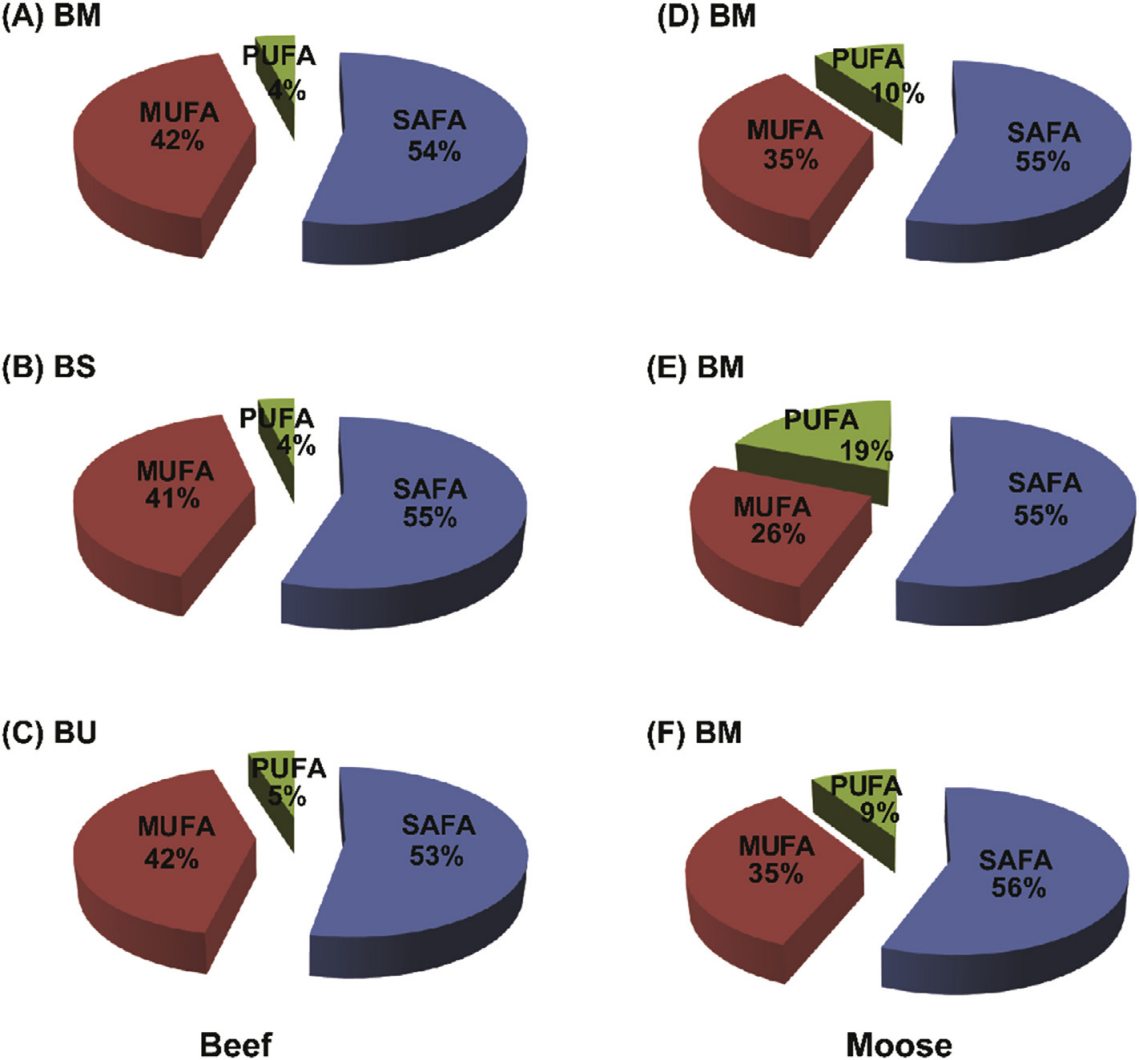
Distribution of fatty acid classes in grilled beef meat. (A–C) represents unmarinated and marinated grilled beef. (D–F) represent unmarinated and marinated moose meat. [BU, MU] = unmarinated grilled beef and moose; [BM, MM] = Indian session ale unfiltered beer-based marinated grilled beef and moose; [BS, MS] = Wheat ale unfiltered beer-based marinated grilled beef and moose. Experimental replication (n) = 3.

**Fig. 2:**
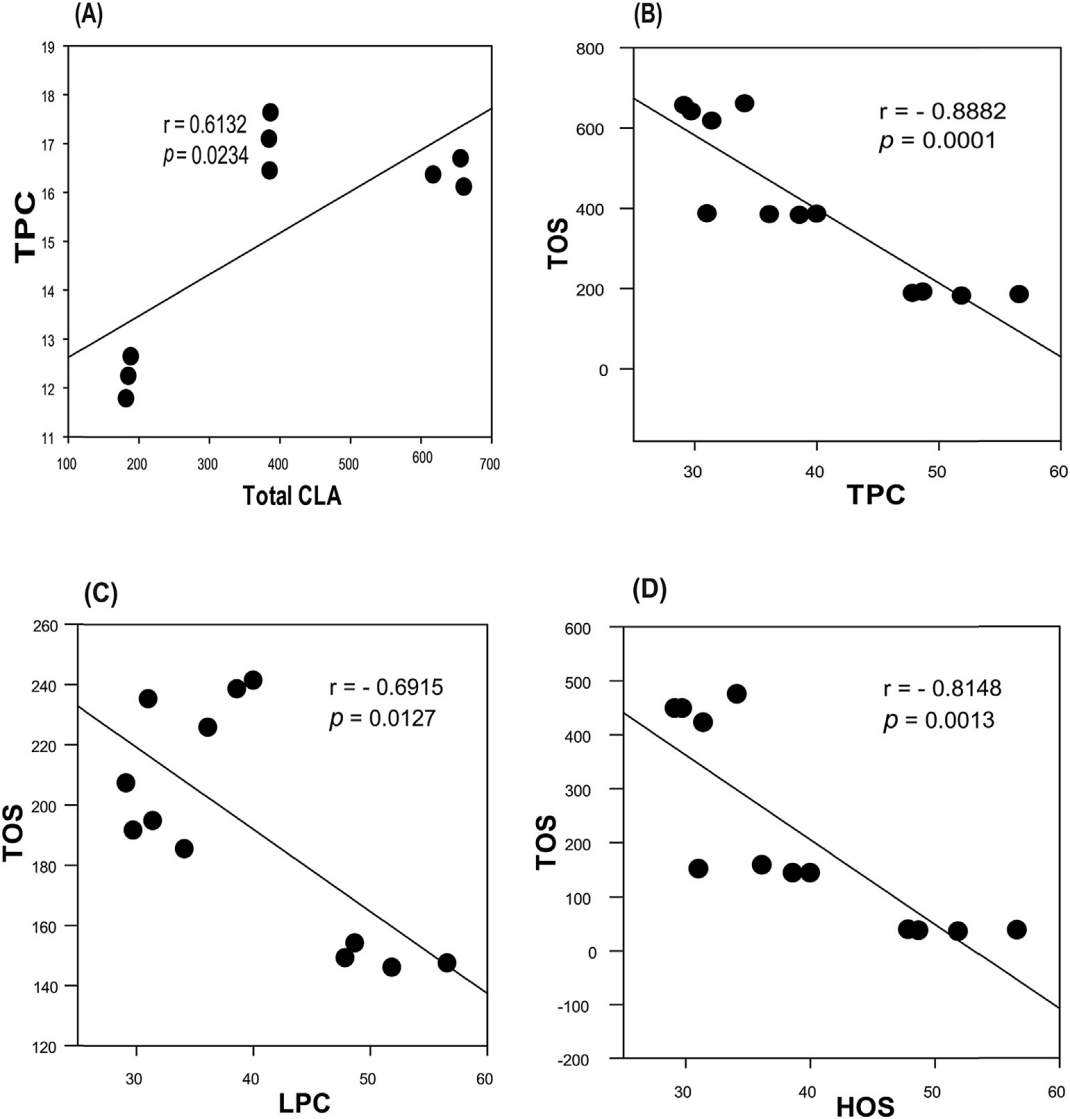
**Pearson's correlation plots for phenolic contents, oxidation status and total CLA content of grilled beef (A**–**D).** R-Values represent Pearson correlation coefficients (r). TPC = HPC + LPC; TOS = HOS + LOS; TPC = Total phenolic content; TOS = Total oxidant status; L = Lipophilic; H = Hydrophilic; CLA = Conjugated linoleic acid. Experimental replication (n) = 3.

**Fig. 3:**
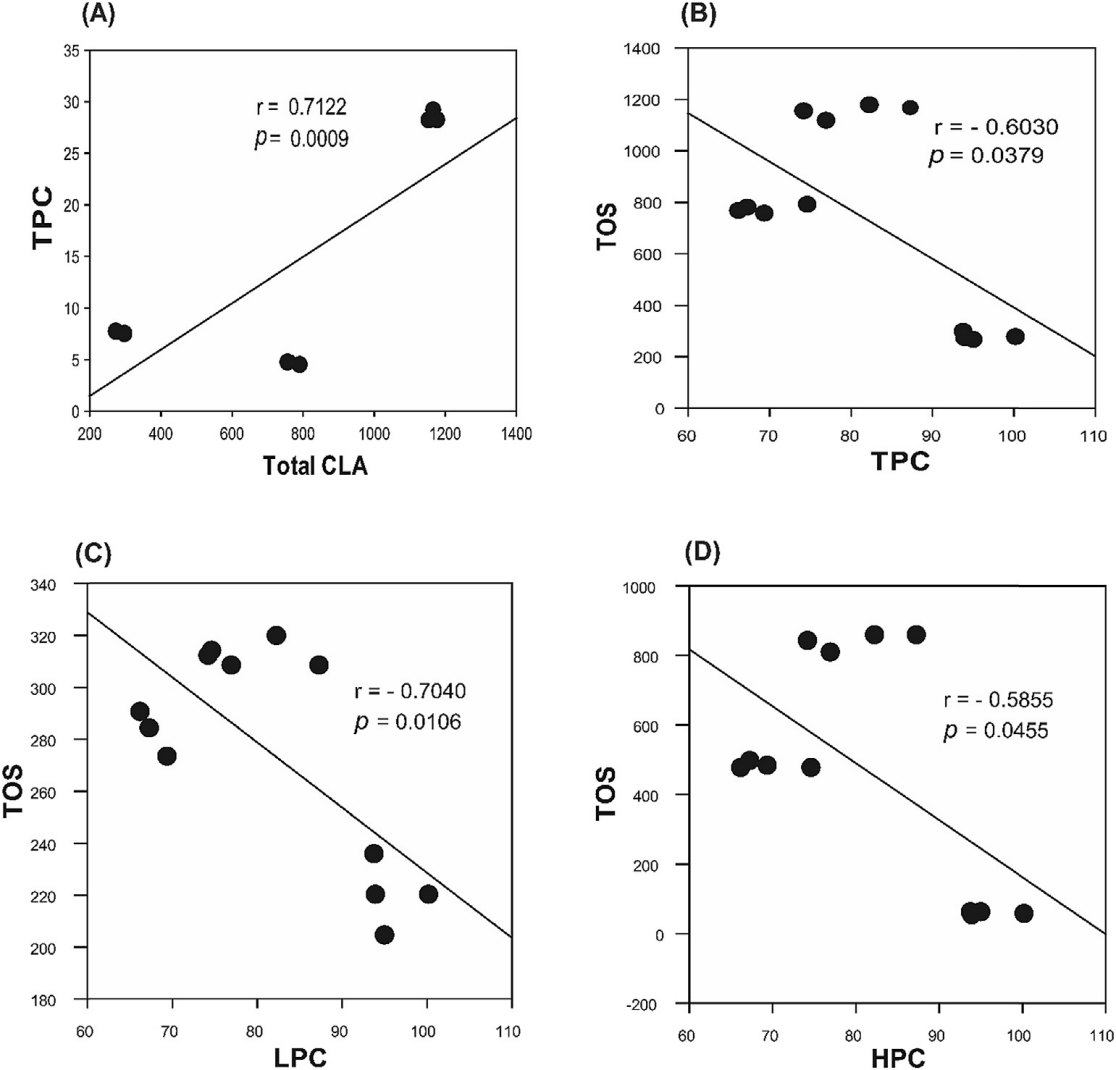
**Pearson's correlation plots for phenolic contents, oxidation status and total CLA content of grilled moose meat (A**–**D).** R-values represent Pearson correlation coefficients (r). TPC = HPC + LPC; TOS = HOS + LOS. TOS = Total oxidant status; TPC = Total phenolic content; L = Lipophilic; H = Hydrophilic; CLA = Conjugated linoleic acid. Experimental replication (n) = 3.

The data set contains 16 fatty acids detected as methyl esters in grilled unfiltered beer based marinated and unmarinated meat samples, and Pearson's correlations plots showing association of total phenolic content and retention of essential anticarcinogenic CLA in the finished products. Table 1 shows the fatty acid composition in grilled beef meat samples; Table 2: the fatty acid composition of grilled moose samples; Table 3: the fatty acid class distribution (mg/g meat) in grilled beef and moose samples; Fig. 1: pie charts showing the percent distribution of fatty acid classes in grilled beef and moose meat; Fig. 2: Pearson's correlations between the total CLA, phenolic contents, and oxidation status in grilled beef samples; and Fig. 3: Pearson's correlations between the total CLA, phenolic contents, and oxidation status in grilled moose samples. The statistical significance between marinated and unmarinated beef and moose samples in terms of their fatty acid compositions is also presented [[2], [3], [4], [5],^7^]. The raw data file is included as supplementary material in this data in brief article.

**Table 1:** Fatty acid composition of grilled unfiltered beer-based marinated and unmarinated beef meats.

Fatty Acid	BM	BS	BU
C10:0	0.47 ± 0.05b	0.55 ± 0.03b	0.27 ± 0.02a
C12:0	0.87 ± 0.03a	8.79 ± 0.33b	0.95 ± 0.02a
C14:0	23.09 ± 0.27a	20.78 ± 0.97a	20.62 ± 1.12a
C15:0	5.42 ± 0.08c	4.74 ± 0.12b	4.08 ± 0.03a
C16:0	131.62 ± 0.52c	125.12 ± 1.37b	115.88 ± 0.64a
C17:0	17.02 ± 0.21c	14.58 ± 0.18b	11.73 ± 0.73a
C18:0	67.60 ± 0.39c	63.54 ± 0.68b	50.46 ± 1.08a
C14:1	5.94 ± 0.07c	5.47 ± 0.03b	4.73 ± 0.17a
C16:1	20.90 ± 0.25b	18.71 ± 0.47a	18.41 ± 0.39a
C17:1	10.22 ± 0.01b	8.30 ± 0.24a	7.70 ± 0.57a
C18:1cis	150.98 ± 1.2c	138.80 ± 1.09b	124.59 ± 4.55a
C20:1	1.83 ± 0.03b	1.71 ± 0.03ab	1.50 ± 0.08a
C18:1n9trans	2.48 ± 0.06a	2.09 ± 0.14a	2.53 ± 0.17a
C18:2n6cis	13.93 ± 0.26a	11.65 ± 0.30a	13.67 ± 0.99a
C18:3n3	2.98 ± 0.07a	3.19 ± 0.07a	3.07 ± 0.12a
C20:4n6	2.04 ± 0.03b	1.46 ± 0.08a	2.30 ± 0.08b

Values represents means ± standard errors; n = 3). Rows with different letters show significant differences between treatments at LSD = 0.05. BU = unmarinated grilled beef; BM = Indian session ale unfiltered beer-based marinated grilled beef; BS = Wheat ale unfiltered beer-based marinated grilled beef.

**Table 2:** Fatty acid composition of grilled unfiltered beer-based marinated and unmarinated moose meats.

Fatty Acid	BM	BS	BU
C10:0	0.47 ± 0.05b	0.55 ± 0.03b	0.27 ± 0.02a
C12:0	0.87 ± 0.03a	8.79 ± 0.33b	0.95 ± 0.02a
C14:0	23.09 ± 0.27a	20.78 ± 0.97a	20.62 ± 1.12a
C15:0	5.42 ± 0.08c	4.74 ± 0.12b	4.08 ± 0.03a
C16:0	131.62 ± 0.52c	125.12 ± 1.37b	115.88 ± 0.64a
C17:0	17.02 ± 0.21c	14.58 ± 0.18b	11.73 ± 0.73a
C18:0	67.60 ± 0.39c	63.54 ± 0.68b	50.46 ± 1.08a
C14:1	5.94 ± 0.07c	5.47 ± 0.03b	4.73 ± 0.17a
C16:1	20.90 ± 0.25b	18.71 ± 0.47a	18.41 ± 0.39a
C17:1	10.22 ± 0.01b	8.30 ± 0.24a	7.70 ± 0.57a
C18:1cis	150.98 ± 1.2c	138.80 ± 1.09b	124.59 ± 4.55a
C20:1	1.83 ± 0.03b	1.71 ± 0.03ab	1.50 ± 0.08a
C18:1n9trans	2.48 ± 0.06a	2.09 ± 0.14a	2.53 ± 0.17a
C18:2n6cis	13.93 ± 0.26a	11.65 ± 0.30a	13.67 ± 0.99a
C18:3n3	2.98 ± 0.07a	3.19 ± 0.07a	3.07 ± 0.12a
C20:4n6	2.04 ± 0.03b	1.46 ± 0.08a	2.30 ± 0.08b

Values represents means ± standard errors; n = 3). Rows with different letters show significant differences between treatments at LSD = 0.05. BU = unmarinated grilled beef; BM = Indian session ale unfiltered beer-based marinated grilled beef; BS = Wheat ale unfiltered beer-based marinated grilled beef.

**Table 3:** Fatty acid classes of grilled unfiltered beer-based marinated and unmarinated beef and moose meats.

Ʃ FA	BM	BS	BU	MM	MS	MU
SAFA	246.09 ± 0.82c	238.09 ± 3.05b	204.00 ± 2.40a	284.31 ± 0.55c	38.08 ± 3.71a	99.93 ± 415b
MUFA	192.36 ± 1.34c	175.08 ± 1.75b	159.47 ± 5.76a	183.18 ± 1.78c	17.68 ± 1.98a	61.1 ± 1.26b
PUFA	18.96 ± 0.36b	16.30 ± 0.41a	19.04 ± 1.19b	51.95 ± 0.29c	13.11 ± 1.08a	16.38 ± 0.36b

Values represents means ± standard errors; n = 3). Rows with different letters show significant differences between treatments at LSD = 0.05. [BU, MU] = unmarinated grilled beef and moose; [BM, MM] = Indian session ale unfiltered beer-based marinated grilled beef and moose; [BS, MS] = Wheat ale unfiltered beer-based marinated grilled beef and moose. FA = Fatty acids; SAFA = Saturated fatty acid; MUFA = Monounsaturated fatty acid; PUFA = Polyunsaturated fatty acids.

## 2 Experimental design, materials, and methods

### 2.1 Reagents and standards

Supelco™ 37 Component FAME mix, CLA methyl ester mix and CLN methyl ester mix were purchased from Sigma Aldrich (St. Louis, USA).

### 2.2 Preparation of marinades

Two types of unfiltered beers were used in this data set: India session ale (M) and Wheat ale (S). Both beers were purchased from a local liquor store in Corner Brook, Newfoundland Canada, and are designated by the manufacturers as unfiltered beers. India session ale contained 4.3% alcohol and was made from water, malted barley, and hops; Wheat ale contained 5.2% alcohol, and was made from water, malted wheat, barley, orange, lemon, lime peel, coriander, Cascade and Willamette hops. To 341 mL of each type of unfiltered beer, a mix of 1 g oregano, 1 g of parsley, 4 g of mustard, 2 g of salt, 8 g of pepper, 1 g of garlic, 25 mL of olive oil, 15 mL of vinegar and 25 g of fresh onions purchased from a local market were added to a food processor and the contents homogenized and mixed thoroughly to obtain the beer-based marinades that was further employed to marinate the moose and beef meat samples [1,6].

### 2.3 Marination of ruminant meat samples

Beef (Bovinae) and moose (ýCervidae) striploin steaks (longissimus muscle) were obtained from a local market and from Newfoundland and Labrador Department of Natural Resources, respectively. Moose steaks were taken from 4 different animals while 4 different beef steak were used to mitigate any inherent variability of the meat source. Ethics approval for this data was granted by Memorial University Animal Care Committee as mandated by the Canadian Council on Animal Care and all the experiments were performed in accordance with relevant guidelines and regulations. Steaks (1 lb) of beef (B) and moose (M) meat from different batches were cut and divided into four replicates (n = 4) per treatment (n = 3). Each replicate was made from an independent batch of beer and ingredients. The steaks were divided into three groups as follow: control group (unmarinated, U), treatment group marinated with India session ale-based marinade (M) and treatment group marinated with Wheat ale beer-based marinade (S). Meat marination was performed by adding 600 mL of each beer-based marinade to the beef and moose steaks for 12 h at 4 °C in zip lock closed plastic bags. The unmarinated samples (U, control) were kept under the same conditions as the marinated ones until time of grilling [1,6].

### 2.4 Cooking conditions

Beef and moose unmarinated (BU, MU) and marinated (BM, BS; MM, MS) samples were grilled at 200–250 °C for 25 min (Cuisinart® Gourmet 600B) reaching an internal temperature of 75 °C. A probe thermometer (Accu-Temp Instant Read Thermometer, model 65613) was used to measure the internal temperature of meat during grilling. In both types of meat, the unmarinated meat was cooked before the marinated ones. The grill was thoroughly cleaned between samples to avoid any possible contamination of marinade flavors. Meat samples were turned regularly during grilling. After grilling, each replicate was divided into two subsets. One subset was cut into two-inch cubes and used for sensory analysis, while the other subset was labeled and stored at −80 ᵒC for chemical analysis [1,6].

### 2.5 Extraction and methylation of meat lipids

Lipids were extracted following the method described by Bligh and Dyer [8]. Briefly, 10 g of sample was mixed with 10 mL of chloroform and 20 mL of methanol, and homogenized with a homogenizer (Tissue Master 125, Omni International, Georgia, U.S.A.) for 2 min. To the mixture, 10 mL chloroform was added and after homogenizing for 2 min, 10 mL of distilled water was added and the mixture vortexed for 2 min. The mixture was filtered through Whatman No. 1 filter paper and the filtrate transferred to a separatory funnel. After allowing the two phases to separate, the bottom layer (organic phase) was collected. Chloroform was removed using a rotary evaporator under reduced pressure at room temperature to avoid meat lipids oxidation. The remaining solvent was removed by evaporating under nitrogen to obtain the fatty acids extracted from the meat samples. The sample was stored at −80 °C until further analysis [1].

An aliquot of the meat lipids (300 μL) was transferred into 2 mL vials along with C18-alkane (final spike concentration 0.01 mg/mL) dissolved in hexane as internal standard. Aliquots (100mL of methanolic-HCl 3 N (Sigma-Aldrich, Ontario, Canada) was added to each sample. The mixtures were then vortexed, followed by incubation in a drying oven at 80 °C for 60 min, removed from the oven and cooled in a fume hood. Distilled water (0.8 mL) was added to the sample and the mixture extracted three times using 500 μL of hexane each time. The hexane fractions were pooled (1.5 mL), and DMP (100 μL) added as a water scavenger, then dried under nitrogen, and the residue re-suspended in 50 μL of hexane. Fatty acid methyl esters (FAMEs) were analyzed by GC-MS [1].

### 2.6 Analysis of fatty acid methyl esters by gas chromatography/mass spectrometry/flame ionisation detector (GC/MS and GC/FID)

GC-MS analysis was conducted on a Thermo Scientific Trace 1300 gas chromatography coupled to a flame ionisation detector and a Thermo Scientific Trace 1300 gas chromatography coupled to a Triple Quad mass spectrometer (Thermo Scientific, Burlington, Ontario, Canada) respectively. Conjugated methylated fatty acids were separated on a BPX70 high resolution column (10 m × 0.1 mm × 0.2 μm; SGE Analytical Science, Victoria, Australia) using helium as the carrier gas at a flow rate of 0.6 mL/min. One microliter (1 μL) of each FAME standard or sample was injected in the system in split mode (15:0) using a Tri-plus auto-sampler. The oven temperature program was as follows: the initial oven temperature 50 °C was held for 0.75 min, then programmed to increase at 4 °C/min to 155 °C, then increased again at 6.0 °C/min to 210 °C, and then increased again at 15 °C/min to finally reach 240 °C and held for 2 min. Identification of the conjugated fatty acids in meat as FAMEs was based on the comparison of their retention times and mass spectra with that of the Supelco™ 37 Component FAME Mix (1 mg/L), CLA and CLN methyl ester standards (Sigma Aldrich Oakville, Ontario, Canada). The amounts of fatty acid identified were calculated and expressed as mg/g meat [1].
